# Development of a simple clinical score to estimate in-hospital adverse event risk after elective hip arthroplasty: a retrospective cohort study in a high-risk population

**DOI:** 10.1007/s00402-025-06128-9

**Published:** 2025-11-18

**Authors:** Matthias Wolf, Dominik Papathanakis, Raphael Trefzer, Christian Merle, Tilman Walker, Julian Deisenhofer

**Affiliations:** 1 Orthopaedic University Hospital, University Hospital Heidelberg, Heidelberg, Germany; 2 Orthopädische Klinik Paulinenhilfe, Diakonie-Klinikum Stuttgart, Stuttgart, Germany

**Keywords:** Arthroplasty, Replacement, Hip, Postoperative complications, Risk assessment, Comorbidity

## Abstract

**Introduction:**

Patients undergoing total hip arthroplasty (THA) in tertiary centres often present with complex comorbidities that increase the risk of perioperative adverse events (AE). While fast-track and outpatient protocols are expanding, reliable risk stratification tools tailored to high-risk European populations remain limited. This study aimed to (1) compare comorbidity burden in a high-risk population to national data, (2) determine incidence and risk factors for in-hospital AE and (3) develop a simple, pragmatic score to identify patients at elevated AE risk.

**Materials and methods:**

We retrospectively analyzed 4,101 elective primary THA cases from a German tertiary care centre (2010–2019). Comorbidity burden was quantified using the Elixhauser Comorbidities (EC) and benchmarked against national registry data (EPRD). Independent predictors of in-hospital AE were identified using multivariate logistic regression. These variables were then used to develop a pragmatic preoperative clinical risk score via LASSO regression, internally validated with 10-fold cross-validation and bootstrapping.

**Results:**

Compared to the national registry, our cohort showed significantly higher rates of major comorbidities, including cardiac valvular disease, diabetes, and fluid/electrolyte disorders. The overall in-hospital AE rate was 2.6%. Six comorbidities—including pulmonary circulation disorders (OR 10.7, 95% CI: 3.6–31.8)—were independently associated with AE. The derived LASSO model demonstrated strong discrimination (AUC 0.80; 95% CI: 0.75–0.84) and calibration (Brier score 0.07). A cutoff score ≥ 2 identified patients with an AE rate of > 7%, while scores < 2 corresponded to an NPV of 0.99, supporting its utility in identifying low-risk patients for fast-track pathways.

**Conclusions:**

Patients treated in tertiary centres exhibit elevated comorbidity burden but maintain acceptable perioperative AE rate. A simple, validated clinical score can flag patients at substantially increased risk of in-hospital AE who may benefit from closer in-hospital surveillance and effectively identify low-risk candidates for fast-track THA pathways. Further external validation is warranted.

## Introduction

Total hip arthroplasty has become a routine procedure that is among the most frequent in Europe and the USA. This frequently includes older patients and those with multiple comorbidities, who remain at elevated risk for perioperative adverse events (AE). Preoperative preparation is key to minimise these risks, however the need for closer clinical and laboratory monitoring often results in prolonged hospital stays, especially in tertiary care centres where patients at increased risk are frequently treated.

In contrast, the current healthcare trend - driven in part by recent policy shifts in Germany - favours fast-track surgical pathways and aims for ambulatory care. While it is widely acknowledged that not all patients are candidates for short-stay protocols, it remains unclear which specific comorbidities or risk factors reliably predict in-hospital AE and thus warrant extended perioperative care.

Several risk stratification tools have been developed to assess perioperative risk. Among the most widely validated are the Elixhauser Comorbidities (EC), which comprise 30 diagnostic categories associated with increased risk of 30-day mortality, and the derivative Elixhauser Index (EI), which converts regression weights into a cumulative score. Additionally, multiple risk stratification tools have been focusing short-stay or outpatient total joint arthroplasty [23]. However, most of these tools are derived from American populations and vary in their endpoints and usability. Some, such as the Outpatient Arthroplasty Risk Assessment (OARA), are proprietary and not readily applicable in European settings [31].

Despite the growing interest in outpatient arthroplasty in Germany, its implementation remains limited. Differences in healthcare systems and patient populations require context-specific tools, particularly in specialised, high-risk centres. In this context, it remains unclear how the baseline comorbidity burden in this high-risk cohort compares to national registries, which factors determine in-hospital AE and if a pragmatic yet simple risk score can estimate AE risk and support fast-track decision-making.

Using data from a decade of elective primary hip arthroplasties at a specialised German academic centre, we aimed to: (1) establish the baseline comorbidity burden in this high-risk population by comparing EC prevalence to the German arthroplasty registry (EPRD); (2) identify independent preoperative risk factors for in-hospital AE; and (3) develop and internally validate a preoperative, pragmatic, yet simple clinical score to estimate AE risk, identifying high-risk patients and possibly supporting shared decision-making around fast-track and outpatient pathways in the future.

## Materials and methods

All patients who underwent elective hip arthroplasty at our centre between 2010 and 2019 were reviewed for inclusion. After data cleaning and a case-by-case assessment of diagnoses and procedures, patients with fractures, tumours or post-infective arthritis of the index joint were excluded to maintain homogeneity within the cohort. Clinical data, including diagnostic codes, procedural details, laboratory values, and mortality records, were collected and organised into a standardised electronic database to enable structured, automated variable classification. In-hospital AE of Clavien-Dindo-Sink grade III or higher were captured [12]. Medical AE requiring inpatient treatment included conditions such as myocardial infarction, pulmonary embolism, renal failure, and deep vein thrombosis, along with their corresponding interventions. Surgical AE comprised any additional procedures involving the index joint, including those for haematomas, infections, or periprosthetic fractures. AE were identified using a structured system based on primary and secondary ICD-10 and OPS codes and case files reviewed individually. Additionally, all coding was subject to routine, case-level quality control by hospital administration, ensuring accurate capture of AE not present at admission. A comprehensive list of relevant diagnostic and procedural codes is provided in Appendix A. Data were structured and analyses performed using Microsoft Excel and Python v3.11.

The preoperative comorbidity profile was standardised using the Elixhauser Comorbidities (EC) [13, 38]. Following statistical consultation, descriptive and exploratory analyses were conducted calculating incidence rates. Intraoperative variables like operative time, surgical approach, drain use and other intraoperative data were not uniformly available and thus were not included in the analyses. To benchmark our cohort, we compared comorbidity prevalence among a subcohort of elective hip arthroplasty patients over 60 to recently published national registry data (EPRD, Endoprothesenregister Deutschland [28]). This dataset uses the same data acquisition methods for diagnoses [28], facilitating comparability.

Univariate logistic regressions of all EC and the known risk factors age and last preoperative mean haemoglobin (Hb) were followed by multivariate logistic regression of significant parameters to determine independent preoperative predictors of AE.

Variables that were independently associated with AE were then entered into a penalised LASSO regression for risk score development. Continuous parameters were standardised. The independently associated EC blood-loss anaemia was replaced by the continuous variable Hb (univariately significant) to better capture the spectrum of anaemia severity instead of a binary variable. Score points were assigned by rounding LASSO regression coefficients and maintaining clinical relevance. For continuous predictors (age and Hb), score brackets were generated in intervals based on standard deviation ranges around the observed means. Model performance was evaluated using AUC (area under the receiver operating characteristic curve), Brier score, and decision curve analysis. Internal validation included 10-fold cross validation during LASSO regression and 1,000-iteration bootstrapping of AUC. Score thresholds and associated performance metrics were derived from the validation data.

## Results

Applying the inclusion criteria, 4,101 elective primary hip arthroplasties were included. The mean age at the time of surgery was 63 ± 14 years (range 15–99), and 56% of procedures were performed in female patients. The average length of hospital stay was 10 ± 5 days (range 1–170).

Intensive care was required in 142 cases (3.5%). Blood transfusions were administered in 953 cases (23.2%). The annual proportion declined from 36.7% in 2010 to 13.1% in 2019. Intraoperative cell salvage was used in 341 cases (8.3%) (see Table 1). The leading indications for arthroplasty were primary osteoarthritis (84.8%), hip dysplasia (9.6%), and avascular necrosis (4.7%). Rare indications were recurrent dislocations (0.5%), and other diagnoses (0.3%).

**Table 1:** Cohort descriptives

	Number/Mean (± SD)	Frequency (%)/Range
Total N	4101	
Age (years)	63 (± 14.1)	
Gender (female)	2316	56.5%
Length of hospital stay (days)	9.9 (± 4.7)	
Cementless fixation (all components)	3352	81.7%
Cemented fixation (any component)	749	18.3%
Hybrid fixation	420	10.2%
ICU treatment (cases)	142	3.46%
Blood transfusions	953	23.24%
Cell salvage	341	8.32%
Revision surgery within hospital stay	8	0.20%
Revisions per case (2010–2019)	155	
1	108	2.63%
2	12	0.29%
3	5	0.12%
4	2	0.05%

*SD* standard deviation, *ICU* intensive care unit, *Hybrid fixation* one component (femoral or acetabular) cemented and the other uncemented, *Cemented “Any component”* includes both hybrid and fully cemented fixation

**Fig. 1:**
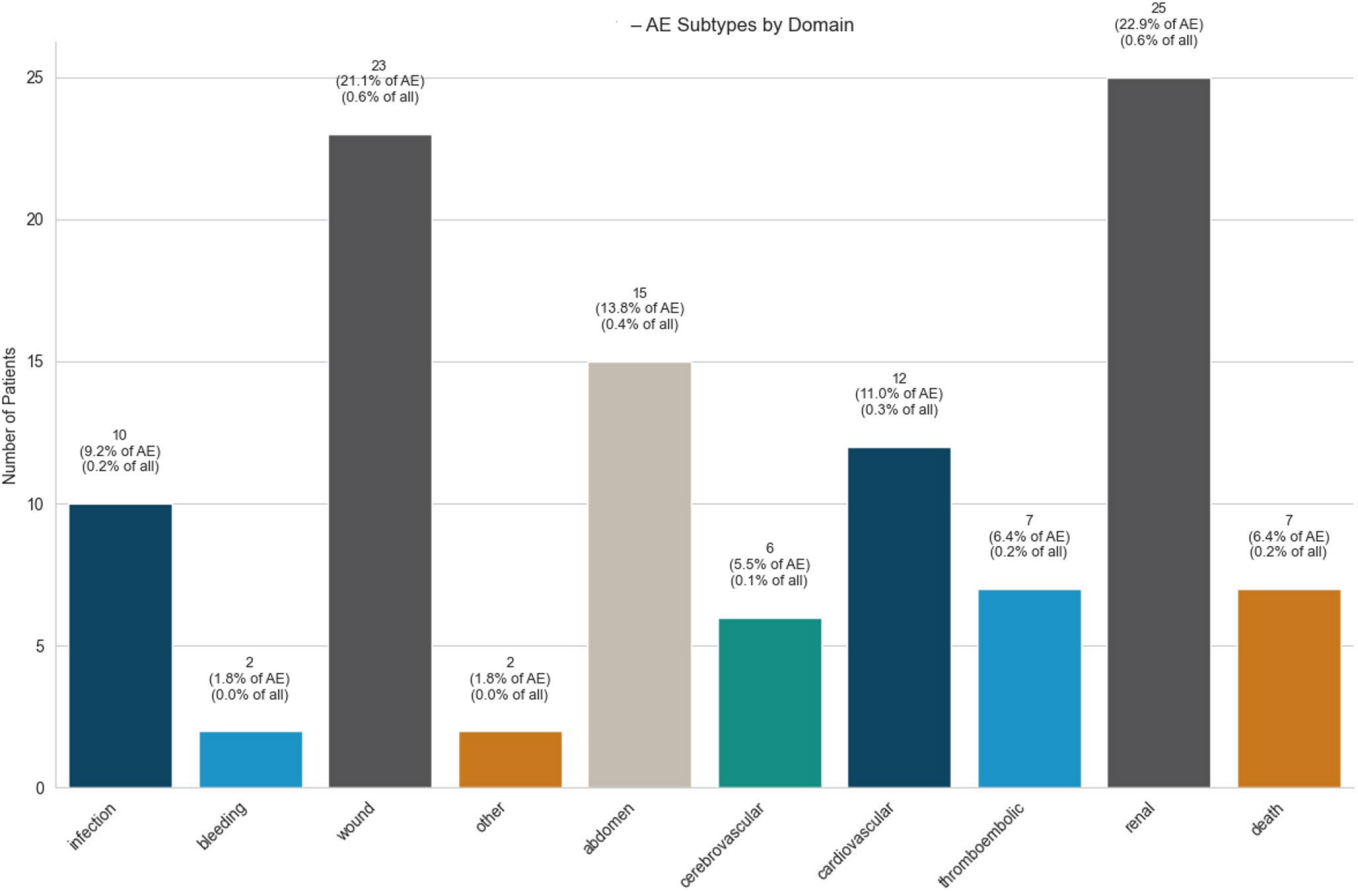
Distribution of adverse events

The overall incidence of in-hospital adverse events (AE) was 2.6% (107 events in 4101 procedures), with 17 patients experiencing multiple AE. Surgical AE accounted for 36% of events, while medical complications accounted for 57% of events. The in-hospital mortality rate was 0.2% (7 cases). A detailed overview of AE is shown in Fig. 1. During the initial hospital stay, 0.2% (8 out of 4,101 surgeries) required revision surgery on the index joint, while 155 in-hospital revision surgeries were recorded within the observed cohort.

Distribution of in-hospital adverse events following elective primary hip arthroplasty. Events are categorised into surgical and major medical complications, including abdominal, cardiovascular, and renal adverse events. Medical complications accounted for the majority of events.

**Fig. 2:**
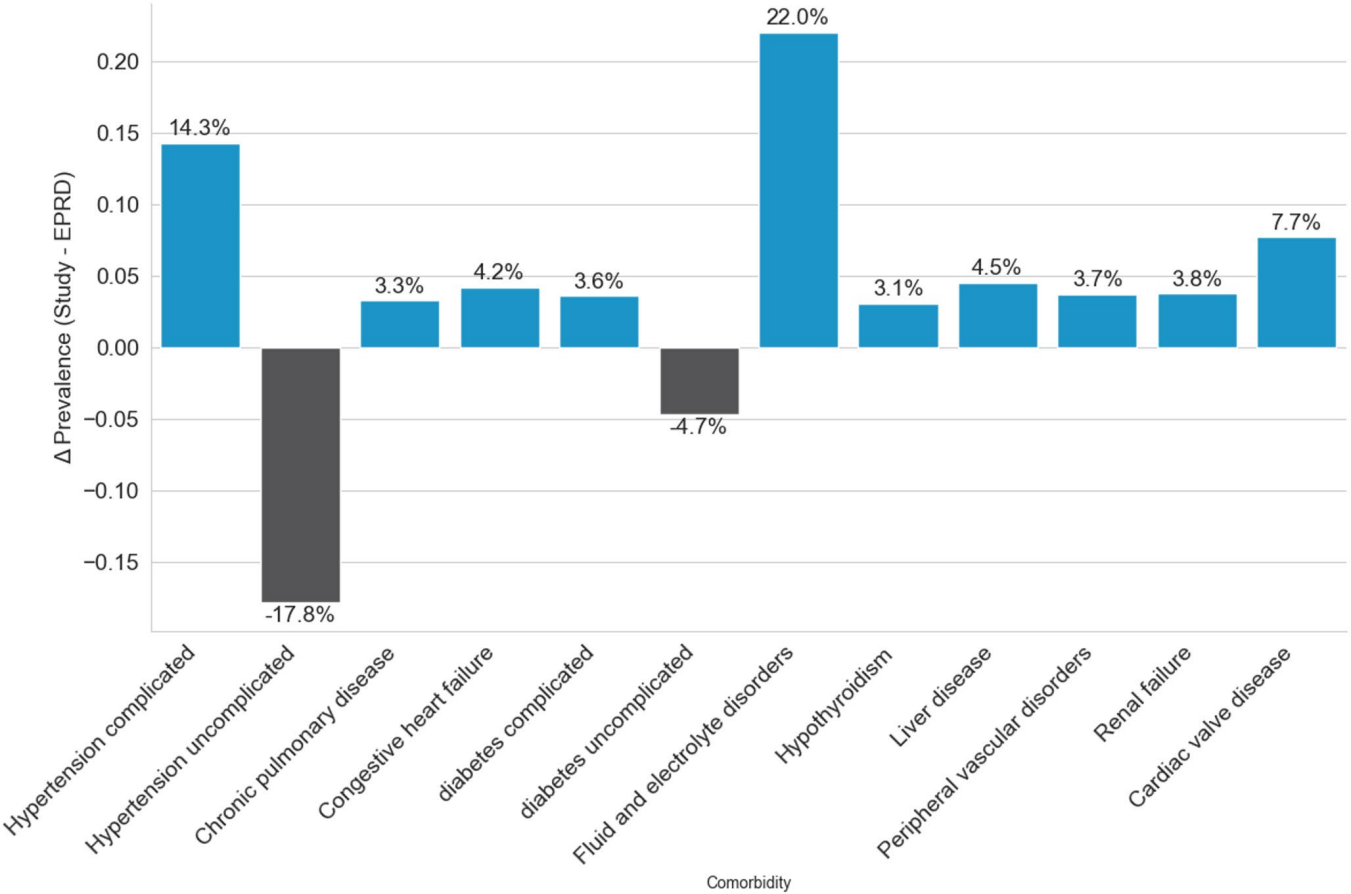
Selected differences in elixhauser comorbidities in comparison to a German registry study

Comparison of comorbidity profiles with a national registry of elective total hip arthroplasty including all patients over the age of 60 [28] revealed significantly higher frequencies of major comorbidities in the study cohort. A statistically significant increase in prevalence of ≥ 3% was observed for complicated arterial hypertension, chronic pulmonary disease, congestive heart failure, complicated diabetes mellitus type 2, fluid and electrolyte disorders, hypothyroidism, liver disease, peripheral vascular disorders, renal failure and cardiac valve disease. This confirmed increased comorbidity burden in national comparison (see Fig. 2 and Appendix B for details). The results were consistent in sensitivity analyses including all study patients.

Only patients over 60 were included in this comparison, only differences ≥ 3% are illustrated. The figure shows an increased prevalence for major comorbidities. See Appendix B for full details.

In univariate logistic regression, both age and last preoperative Hb values were significantly associated with in-hospital AE (*p* < 0.001 for both). Among the 31 Elixhauser Comorbidity categories, 12 were significantly associated with AEs. Multivariate logistic regression identified the following independent predictors of in-hospital AEs: blood-loss anaemia, coagulopathy, congestive heart failure, fluid and electrolyte disorders, pulmonary circulation disorders and renal failure. Pulmonary circulation disorders were the strongest predictor, with an odds ratio (OR) of 10.7 (95% CI: 3.6–31.8) (See Table 2).

**Table 2:** Predictive factors of adverse events

	total		univariate analysis			binary log. regression		
	mean(SD)	(95% CI)	*N* (AE)	% (AE)	Sig.	sig.	OR	95% CI
Age	63 (14.1)	30–85			< 0.001	**0.035**	**1.02**	**1.00–1.05**
Last preop. Hb value	13.8 (1.4)	11–16			< 0.001	0.402*	1.08	0.9–1.3
	**n (no AE)**	**% (no AE)**						
AIDS/HIV	3	0.1%	0	0.0%	1.000			
Alcohol abuse	72	1.8%	3	0.1%	0.214			
Bloodloss anaemia	1081	26.4%	54	1.3%	0.000	**0.000**	**3.57**	**2.02–6.30**
Complicated arterial hypertension	447	10.9%	27	0.7%	0.000	0.566	1.21	0.63–2.32
Uncomplicated arterial hypertension	1561	38.1%	29	0.7%	0.580			
Cardiac arrhythmias	432	10.5%	16	0.4%	0.012	0.361	0.71	0.34–1.48
Chronic pulmonary disease	379	9.2%	15	0.4%	0.007	0.069	1.86	0.95–3.61
Coagulopathy	140	3.4%	16	0.4%	0.000	**0.010**	**2.61**	**1.26–5.40**
Congestive heart failure	195	4.8%	20	0.5%	0.000	**0.050**	**2.09**	**1.00–4.39**
Deficiency anaemia	20	0.5%	1	0.0%	0.374			
Depression	185	4.5%	4	0.1%	0.886			
Complicated Diabetes Mellitus	143	3.5%	6	0.1%	0.074			
Uncomplicated Diabetes Mellitus	291	7.1%	8	0.2%	0.368			
Drug abuse	17	0.4%	1	0.0%	0.297			
Fluid and electrolyte disorders	1285	31.3%	48	1.2%	0.000	**0.002**	**2.27**	**1.36–3.81**
Hypothyroidism	600	14.6%	7	0.2%	0.121			
Liver disease	204	5.0%	5	0.1%	0.657			
Lymphoma	31	0.8%	1	0.0%	0.642			
Metastatic cancer	21	0.5%	0	0.0%	1.000			
Obesity	602	14.7%	14	0.3%	0.565			
Other neurological disorders	94	2.3%	6	0.1%	0.006	0.076	2.62	0.90–7.58
Paralysis	59	1.4%	3	0.1%	0.116			
Peptic ulcer disease	7	0.2%	0	0.0%	0.994			
Peripheral vascular disorders	179	4.4%	7	0.2%	0.079			
Psychosis	18	0.4%	1	0.0%	0.323			
Pulmonary circulation disorders	19	0.5%	11	0.3%	0.000	**0.000**	**10.68**	**3.58–31.82**
Renal failure	360	8.8%	27	0.7%	0.000	**0.043**	**1.96**	**1.02–3.76**
Rheumatoid diseases	166	4.0%	5	0.1%	0.366			
Solid tumour no metastasis	58	1.4%	4	0.1%	0.017	0.117	2.52	0.79–8.00
Valvular disease	277	6.8%	16	0.4%	0.000	0.338	1.45	0.68–3.11
Weight loss	4	0.1%	0	0.0%	1.000			

*n* number, *Sig.* significance, *OR* odds ratio, *Diff.* difference, *AIDS/HIV* acquired immunodeficiency syndrome/human immunodeficiency virus infection. Univariate and multivariate logistic regressions were performed. Bold values are significant at *p* < 0.05

*Hb was not independently associated with AE but included into the LASSO model replacing blood-loss anaemia for clinical considerations

A penalised LASSO regression model was used to develop a clinical risk score. Although last preoperative haemoglobin did not remain independently significant in multivariate analysis, it replaced the EC category of blood-loss anaemia in the LASSO model to better reflect the continuous spectrum of anaemia. Standardised regression coefficients were rounded to assign score points. Age and haemoglobin were binned into clinically meaningful intervals based on standard deviation ranges around cohort means. Coefficients corresponding to the centre of each bin were the basis for the resulting score structure (see Table 3).

**Table 3:** Results of the LASSO regression

Parameters		Coefficient	Score points
Pulmonary circulation disorders		4.43	4
Coagulopathy		2.19	2
Renal failure		0.80	1
Fluid and electrolyte disorders		0.83	1
Age (mean 64.4, standard deviation 12.8)*		0.53	0
20	35	−1.33	−1
36	50	−0.77	−1
51	65	−0.21	0
66	75	0.26	0
76	90	0.73	1
90	99	1.18	1
Hb (mean 13.8, standard deviation 1.4)*		−0.38	0
9.7	11.1	0.94	1
11.2	12.4	0.57	1
12.5	13.8	0.19	0
13.9	15.2	−0.19	0
15.3	16.6	−0.57	−1
16.7	18.0	−0.94	−1

*Continuous variables were binned symmetrically around the mean, with each score point reflecting the LASSO coefficient at the centre of the bin. For example, the age bin 51.6–64.4 corresponds to one standard deviation above the mean, resulting in a coefficient scaled to 0.5 of the original LASSO estimate. *N* = 3,729 cases were included in the LASSO regression based on availability of last preoperative haemoglobin

**Fig. 3:**
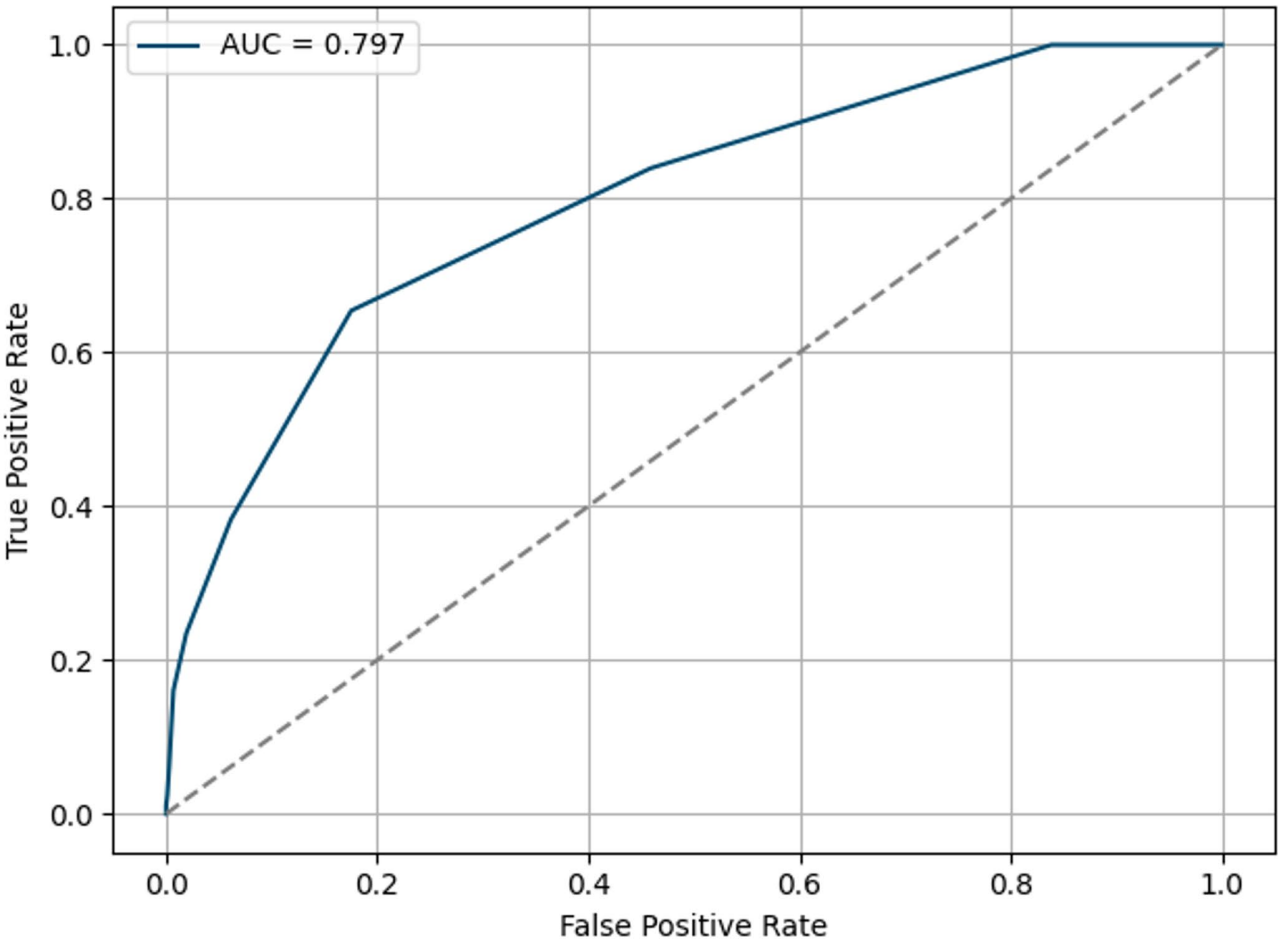
Bootstrapped ROC curve for LASSO-derived risk score

The final LASSO model showed strong discrimination and calibration, with a bootstrapped AUC of 0.80 (95% CI: 0.75–0.84) and a Brier score of 0.07 (95% CI: 0.06–0.103) (see Fig. 3). Threshold analysis identified an optimal cut-off score of ≥ 2 based on Youden’s Index and specificity considerations. At this threshold, the model achieved a specificity of 0.82, sensitivity of 0.65, positive predictive value (PPV) of 0.07, and negative predictive value (NPV) of 0.99. Patients scoring ≥ 2 had an AE rate of 7%, whereas those below this threshold had an AE rate near the cohort average. Thus, the score performs best in identifying low-risk individuals suitable for standard or fast-track perioperative care (high NPV) (see Appendix C).

Receiver operating characteristic (ROC) curve illustrating the discriminative performance of the LASSO-based clinical risk score for predicting in-hospital adverse events. The curve reflects 1,000 bootstrap iterations, yielding a mean AUC of 0.80 (95% CI: 0.75–0.84), indicating good model discrimination.

## Discussion

The use of the Elixhauser Comorbidity (EC) system, along with the ICD-10 adaptation [38, 41], allows for robust comparisons across patient cohorts [13, 41] and has been widely validated in orthopaedic settings as a predictor of in-hospital mortality and perioperative risk [13, 32]. Our analysis revealed a notably higher prevalence of key comorbidities among elective arthroplasty patients over 60 at a German tertiary care centre compared to a national registry (EPRD) [28]. Unfortunately there were no data available from the EPRD to compare the entire study cohort, albeit the findings remained stable when comparing all patients of the study to patients over 60 in the EPRD. These findings confirm the expectation that tertiary referral centres manage more medically complex populations. Despite this elevated risk profile, the observed in-hospital AE rate of 2.6% compares favourably with international benchmarks. For example, an Australian multicentre registry reported a 3% in-hospital complication rate for major events, and the NSQIP database has shown 30-day major complication rates of 4.2% for total hip arthroplasty (THA) [4, 35]. Although direct comparison is complicated by differing follow-up durations, the 10-day mean length of stay in our cohort ensures that a substantial proportion of early complications would be captured. Reported in-hospital mortality in our study (0.2%) also falls within the internationally reported range of 0.05% to 1.1%, depending on population age and postoperative interval [3, 5, 21, 25, 40]. Among patients aged 80–90 years, mortality can exceed 2.5%, underscoring the importance of robust preoperative risk assessment and stratification in high-risk populations [33].

This retrospective analysis used variables consistently available before incision; detailed intraoperative factors possibly associated with adverse events (operative time, approach distribution, and the utilisation of tranexamic acid or drains [22, 34, 37]) were not captured uniformly at cohort level and were not analysed. Procedures were undertaken by fellowship-trained arthroplasty surgeons to national standards, using a minimally invasive anterolateral approach or modified lateral approach; antimicrobial prophylaxis followed a single-dose cefuroxime policy. Drain use decreased over the decade and was generally avoided by the end, although individual frequencies are not available.

Transfusion utilisation in this cohort was higher than some contemporary reports from high-volume or international centres during the same period [7, 29]. This should be interpreted in the setting of a comorbidity-enriched tertiary case-mix and a timeframe in which patient blood-management programmes were being introduced. Consistent with that transition, transfusion use declined substantially across the study window, and similarly high rates had been reported before patient-blood management implementation [19]. Given recognised links between anaemia, transfusion exposure, postoperative infection risk and acute kidney injury [10, 15, 24], last preoperative haemoglobin was included into the score as a continuous preoperative marker of anaemia severity although it was not significant in multivariate analysis. It replaced blood-loss anaemia, which itself was independently associated with AE.

Cardiopulmonary comorbidities were central to risk prediction in our model, reflecting the systemic physiologic stress of arthroplasty. Surgical blood loss, immobility, thromboembolic risk, and systemic inflammatory response all increase vulnerability in patients with preexisting cardiovascular disease. The independent predictive value of congestive heart failure, coagulopathy, and fluid/electrolyte disorders mirrors findings from U.S. datasets and perioperative risk models [14, 30, 32]. This is reflected in U.S. cardiology guidelines that emphasise functional status and cardiac comorbidities in perioperative evaluation [16]. Notably, despite structured preoperative assessment at our institution, these comorbidities retained independent predictive value for AE.

Fluid and electrolyte disorders demonstrated low effect sizes, while sodium imbalances have been associated with AE in prior studies [42]. These findings may reflect successful preoperative optimisation, patient selection biases, or the treatable nature of certain comorbidities. It may also reflect chronic kidney disease, which is a known risk factor for adverse outcomes [1, 10, 24].

Pulmonary circulation disorders emerged as the most powerful predictor of AE in this study. While prior studies have linked these disorders to postoperative complications and VTE-related mortality [39], our findings suggest a broader association with overall AE risk. For instance, one study noted elevated complication rates among patients with pulmonary artery systolic pressures exceeding 35 mmHg [26]. While rigorous haemodynamic definitions (e.g., mean pulmonary artery pressure >25 mmHg via right heart catheterisation) may yield even greater risk estimates, our findings further support the prognostic importance of this comorbidity.

Multiple risk prediction tools for readmission, discharge disposition, or complications following joint arthroplasty have been summarised in a systematic review by Howie et al. [23]. Examples include the Total Joint Replacement Risk Calculator (AJRR) [9], ACS Risk Calculator [6], Duke Readmission Calculator [18], OrthoCincy Readmission Tool [20], and the Readmission Risk Assessment Tool [8], some of which utilise Elixhauser comorbidities. Discharge calculators such as the Iowa Discharge Disposition Score [17], Cleveland Clinic Calculator [2], and the Australian-based Risk Assessment and Prediction Tool [36] have also been described. The Penn Arthroplasty Risk Score specifically assesses perioperative risk for intensive care admission [11]. The widely cited Outpatient Arthroplasty Risk Assessment(OARA) Score is considered among the most sophisticated tools, focusing on predicting successful same-day or short-stay discharge [31]. Although validated in follow-up studies [27, 44], the proprietary nature of the OARA score limits reproducibility and introduces potential bias, particularly for institutions without access to such proprietary resources like many in Europe.

A further limitation of these tools is their reliance on U.S. patient populations, which differ substantially from cohorts in European tertiary centres, as previously demonstrated for primary shoulder arthroplasty patients [43]. Such differences restrict interpretability and practical application within European and specifically German healthcare systems due to varying system structures and financial constraints associated with proprietary tools.

Moreover, these existing calculators generally require extensive variable input from differing sources, making them impractical in everyday practice and high-volume clinical settings such as the present study centre. In contrast, the present study aimed to create a simpler, questionnaire-based risk assessment based on medical history and laboratory testing alone to facilitate clinical implementation.

Finally, a major limitation shared by these tools—and the current study—is the absence of external validation, initially restricting generalizability beyond the originating institution.

A score cut-off of ≥ 2, selected based on Youden’s index and specificity considerations, identified a subset of patients with a minimum 7% AE rate and high negative predictive value (0.99). Patients with scores < 2 experienced AE rates near the cohort average and may possibly be safely considered for fast-track care with standard monitoring. For outpatient arthroplasty, even stricter criteria should be applied. In our study, patients with scores < 1 had the lowest AE rate observed with ~ 2% and thus could potentially be eligible for such pathways. In summary, the data suggest that risk score thresholds may aid clinicians in determining eligibility for short-stay or outpatient protocols. Nevertheless, these findings must be interpreted cautiously within similar highly specialised and comorbidity-enriched settings in the absence of external validation on wider, more general cohorts.

## Strengths and limitations

This study provides a detailed assessment of perioperative outcomes in a large, high-risk cohort of 4,101 primary hip arthroplasty patients at a German tertiary care centre. The use of standardised EC classification enables direct comparison with national and international datasets. AE capture through billing and structured electronic data extraction minimised interpretation bias, particularly for major events with reimbursement implications.

However, the study is limited by its single-centre design, which excludes generalisability across healthcare systems pending external validation, albeit internal validation was rigorous. In this retrospective study, intraoperative data (e.g. operative time, number of surgeons, drain use, tranexamic-acid usage) were not available at cohort level and thus excluded from description and analyses, introducing potential bias from omitted intraoperative variables. Practice changes across the study period may also contribute to secular trend effects, which were not the focus of the present analysis. The absence of long-term follow-up further precludes conclusions regarding prosthetic survivorship or late complications, which were not the aim of the study. Nonetheless, the exclusive focus on in-hospital events offers a clean view of perioperative safety, free from confounding by unrelated later complications.

## Conclusion

This study highlights the increased comorbidity burden of patients undergoing arthroplasty at a tertiary care centre in Germany, relative to national registry benchmarks. Despite this, perioperative AE and mortality rates remained well within international standards. Pulmonary circulation disorders emerged as the most robust predictor of adverse events, followed by multiple cardiopulmonary comorbidities. These findings underscore the importance of comprehensive preoperative evaluation and multidisciplinary care.

Based on these findings, we developed a pragmatic, internally validated clinical risk score to estimate AE risk. The score demonstrated strong discrimination and calibration and may serve as a practical tool to support preoperative decision-making. Its clinical advantage lies in flagging patients at substantially increased risk of in-hospital AE who may benefit from closer in-hospital surveillance, while its statistical strength supports the safe selection of low-risk candidates for fast-track pathways. External validation is planned to assess the generalisability of this approach and to confirm its utility.

## Data Availability

The datasets used and/or analysed during the current study are available from the corresponding author on reasonable request.

## Appendix A

Extended list and procedural file for adverse event classification.

**Table:**

Category	ICD_Codes
Symptoms and signs involving the digestive system and abdomen	R10.0, R10.1, R10.2, R10.3, R10.4
Vascular disorders of the intestine	K55.0, K55.1, K55.21, K55.22, K55.31, K55.8
Complications of surgical and medical care, not elsewhere classified	T81.0, T81.1, T81.2, T81.3, T81.4, T81.5, T81.6, T81.7, T81.8, T81.9
Pyogenic arthritis	M00.00, M00.01, M00.02, M00.03, M00.04, M00.05, M00.06, M00.07, M00.08, M00.09, M00.10, M00.11, M00.12, M00.13, M00.14, M00.15, M00.16, M00.17, M00.18, M00.19, M00.20, M00.21, M00.22, M00.23, M00.24, M00.25, M00.26, M00.27, M00.28, M00.29, M00.80, M00.81, M00.82, M00.83, M00.84, M00.85, M00.86, M00.87, M00.88, M00.89, M00.90, M00.91, M00.92, M00.93, M00.94, M00.95, M00.96, M00.97, M00.98, M00.99
Cutaneous abscess, furuncle and carbuncle	L02.0, L02.1, L02.2, L02.3, L02.4, L02.8, L02.9
Internal derangement of the knee	M23.50, M23.51, M23.52, M23.53, M23.54, M23.57, M23.59, M23.60, M23.61, M23.62, M23.63, M23.64, M23.67, M23.69, M23.80, M23.81, M23.82, M23.83, M23.84, M23.87, M23.89, M23.90, M23.91, M23.92, M23.93, M23.94, M23.95, M23.96, M23.97, M23.99
Complications of internal orthopaedic prosthetic devices, implants and grafts	T84.00, T84.01, T84.02, T84.03, T84.04, T84.05, T84.06, T84.07, T84.08, T84.09, T84.10, T84.11, T84.12, T84.13, T84.14, T84.15, T84.16, T84.18, T84.20, T84.28, T84.3, T84.4, T84.5, T84.6, T84.7, T84.8, T84.9
Other disorders of the muscle	M62.40, M62.41, M62.42, M62.43, M62.44, M62.45, M62.46, M62.47, M62.48, M62.49
Phlebitis and thrombophlebitis	I80.0, I80.1, I80.20, I80.28, I80.3, I80.80, I80.81, I80.88, I80.9

To begin the analysis, an initial review of the data to identify the different complications present within the dataset was conducted - this process involved systematically filtering and categorising all available secondary diagnosis of the dataset, stepwise searching for specific ICD codes associated with various medical complications, allowing to determine which types of complications were recorded – and how.

Following this, the data was scanned for potential subcategories within these complications. Subcategories were identified based on more detailed aspects of the complications -including its clinical relevance- and were then used to conduct a more in-depth statistical analysis, resulting in the structure provided in Table 1 of the manuscript. This step ensured that the analysis captured both the broader categories and the specific nuances within each complication type associated with orthopaedic surgery. Using this method around 15000 possible ICD/OPS codes were narrowed down to 197 codes that were present within the dataset and defined as AE, leading to the following systematic:

**Table:**

Feature	Codes (ICD/OPS)
AE_ICD_abd_acute	R10.0
AE_ICD_abd_vessel_occlusion	K55.0
AE_ICD_blutung_durchOP	T81.1
AE_ICD_fremdk_durchOP	T81.5
AE_ICD_haematom_bleeding_durchOP	T81.0
AE_ICD_iatrwu, AEe_durchOP	T81.2
AE_ICD_inf_durchOP	T81.4
AE_ICD_periop_other_durchOP	T81.8, T81.9
AE_ICD_periop_vasc_durchOP	T81.7
AE_ICD_TVT	I80.0, I80.1, I80.20, I80.28, I80.3, I80.80, I80.81, I80.88, I80.9
AE_ICD_WHS_durchOP	T81.3
AE_ICD_abd_ileus	K56.0, K56.3, K56.5
AE_ICD_abd_ileus_other	K56.6, K56.7, K56.4
AE_ICD_Herzstillstand_ROSC	I46.0
AE_ICD_Herzstillstand_Tod	I46.1, I46.9
AE_Cerebralhemmorrhage	I60., I60.0, I60.1, I60.2, I60.3, I60.4, I60.5, I60.6, I60.7, I60.8, I60.9, I61., I61.0, I61.1, I61.2, I61.3, I61.4, I61.5, I61.6, I61.8, I61.9, I62., I62.0, I62.00, I62.01, I62.02, I62.09, I62.1, I62.9
AE_ICD_LAE	I26.0, I26.9
AE_ICD_Myokardinfarkt	I21.0, I21.1, I21.2, I21.3, I21.4, I21.40, I21.41, I21.42, I21.48, I21.9
AE_Renalinsufficiency_acute	N17.0, N17.01, N17.02, N17.03, N17.09, N17.1, N17.11, N17.12, N17.13, N17.19, N17.2, N17.21, N17.22, N17.23, N17.29, N17.8, N17.81, N17.82, N17.83, N17.89, N17.9, N17.91, N17.92, N17.93, N17.99
AE_ICD_Stroke	I63., I63.0, I63.1, I63.2, I63.3, I63.4, I63.5, I63.6, I63.8, I63.9, I64
AE_OP_abd_laparotomy	5-541.0, 5-541.1, 5-541.2, 5-541.3, 5-541.4, 5-541.5, 5-541.6, 5-541.x, 5-541.y
AE_OP_vasc_surgical_, abdpelvother	5-389.5x
AE_OP_vasc_surgical_, AKneeOther	5-389.7x
AE_OP_vasc_surgical_, Aproffem	5-389.71
AE_OP_vasc_surgical_, vascother	5-389.ax, 5-389.x, 5-389.y
AE_OP_Vesselreconstruction_other	5-388.70, 5-388.9B, 5-395.70, 5-394.1
AE_OP_closureartery	5-389.5X, 5-389.71, 5-389.7X, 5-389.X
AE_OP_closurevein	5-389.A5, 5–389.9 K
AE_ICD_CPR	8-779

## Appendix B

Overview of Adverse Events, comparison to a national German registry cohort.

**Table:**

	Total		Comparison to EPRD 2023			
	*n* (of 4101)	% (total)	Diff.%	relative	*n* (of 263967)	% (EPRD)
AIDS/HIV	3	0.1%	0.1%	38.6	5	0.0%
Alcohol abuse	75	1.8%	1.6%	7.3	658	0.2%
Bloodloss Anaemia	1138	27.7%				
**Complicated arterial hypertension**	**474**	**11.6%**	**8.9%**	**4.4**	**6964**	**2.6%**
**Uncomplicated arterial hypertension**	**1590**	**38.8%**	**-25.7%**	**0.6**	**170176**	**64.5%**
Cardiac arrhythmias	448	10.9%	-2.1%	0.8	34310	13.0%
Chronic pulmonary disease	394	9.6%	1.5%	1.2	21463	8.1%
Coagulopathy	156	3.8%	2.3%	2.5	4054	1.5%
Congestive heart failure	215	5.2%	1.9%	1.6	8811	3.3%
Deficiency anaemia	21	0.5%	-0.6%	0.5	2982	1.1%
Depression	189	4.6%	0.3%	1.1	11397	4.3%
Complicated Diabetes Mellitus	149	3.6%	1.8%	2.0	4724	1.8%
**Uncomplicated Diabetes Mellitus**	**299**	**7.3%**	**-6.3%**	**0.5**	**35783**	**13.6%**
Drug abuse	18	0.4%	0.3%	3.0	381	0.1%
**Fluid and electrolyte disorders**	**1333**	**32.5%**	**23.0%**	**3.4**	**25119**	**9.5%**
Hypothyroidism	607	14.8%	1.7%	1.1	34508	13.1%
**Liver disease**	**209**	**5.1%**	**4.3%**	**6.6**	**2040**	**0.8%**
Lymphoma	32	0.8%	0.7%	6.0	343	0.1%
Metastatic cancer	21	0.5%	0.4%	3.2	420	0.2%
Obesity	616	15.0%	-0.9%	0.9	41951	15.9%
Other neurological disorders	100	2.4%	0.8%	1.5	4372	1.7%
Paralysis	62	1.5%	1.0%	3.0	1317	0.5%
Peptic ulcer disease	7	0.2%	0.1%	5.1	88	0.0%
Peripheral vascular disorders	186	4.5%	2.1%	1.8	6503	2.5%
Psychosis	19	0.5%	0.3%	3.0	412	0.2%
Pulmonary circulation disorders	30	0.7%	0.2%	1.3	1461	0.6%
Renal failure	387	9.4%	0.2%	1.0	24275	9.2%
Rheumatoid diseases	171	4.2%	1.2%	1.4	7735	2.9%
Solid tumour no metastasis	62	1.5%	1.0%	2.7	1455	0.6%
**Valvular disease**	**293**	**7.1%**	**4.3%**	**2.5**	**7589**	**2.9%**
Weight loss	4	0.1%	-0.9%	0.1	2682	1.0%

n – number, Sig. – sigma, OR – odds ratio, Diff. – difference, AIDS/HIV – acquire immune deficiency syndrome/human immunodeficiency virus infection.

The cohort was compared to a national registry as published by Leopold. Krull et al., JBJS 2023.

Negative values in Difference % reflect a more prevalent frequency in the German national registry cohort (EPRD, Endoprothesenregister Deutschland). Relative differences report the increase or decrease observed in the present study in relation to the EPRD. Diff% and relative are printed in bold for variables significant at *p* < 0.005 in chi-square test of fisher’s exact test, depending on minimal counts.

## Appendix C

Performance Metrics by Risk Score Cut-Off.

## Abbreviations

AE: Adverse Event(s)
aHT: Arterial Hypertension
CI: Confidence Interval
EC: Elixhauser Comorbidity/Comorbidities
EPRD: Endoprothesenregister Deutschland (German Arthroplasty Registry)
Hb: Haemoglobin
ICU: Intensive Care Unit
LASSO: Least Absolute Shrinkage and Selection Operator
NPV: Negative Predictive Value
OPS: Operationen- und Prozedurenschlüssel (German procedure code)
OR: Odds Ratio
PPV: Positive Predictive Value
ROC: Receiver Operating Characteristic
SD: Standard Deviation
THA: Total Hip Arthroplasty
